# Long non-coding RNA HULC as a novel serum biomarker for diagnosis and prognosis prediction of gastric cancer

**DOI:** 10.18632/oncotarget.10107

**Published:** 2016-06-16

**Authors:** Chunjing Jin, Wei Shi, Feng Wang, Xianjuan Shen, Jing Qi, Hui Cong, Jie Yuan, Linying Shi, Bingying Zhu, Xi Luo, Yan Zhang, Shaoqing Ju

**Affiliations:** ^1^ Medical School of Medicine, Nantong University, Nantong 226000, Jiangsu Province, China; ^2^ Surgical Comprehensive Laboratory, Affiliated Hospital of Nantong University, Nantong 226000, Jiangsu Province, China; ^3^ Laboratory Medicine Center, Affiliated Hospital of Nantong University, Nantong 226000, Jiangsu Province, China

**Keywords:** gastric cancer, long non-coding RNA, biomarker, HULC

## Abstract

Long non-coding RNAs (lncRNAs) have recently emerged as vital players in tumor biology with potential value in cancer diagnosis, prognosis, and therapeutics. The lncRNA HULC (highly up-regulated in liver cancer) is increased in many malignancies, yet its serum expression profile and clinical value in gastric cancer (GC) patients remain unclear. Quantitative real-time polymerase chain reaction (RT-qPCR) for large-scale analysis of the serum expression of HULC in GC patients reliably detected circulating HULC and revealed that it is upregulated in GC patients. A high serum HULC level correlated with tumor size, lymph node metastasis, distant metastasis, tumor-node-metastasis stage, and *H. pylori* infection. The area under the ROC curve for HULC was up to 0.888, which was higher than that for CEA (0.694) and CA72-4 (0.514). Follow-up detection and Kaplan-Meier curve analysis revealed HULC is a good predictor of GC prognosis. Our present study indicates that circulating HULC may represent a novel serum tumor marker for early diagnosis and monitoring progression and prognosis of GC.

## INTRODUCTION

Gastric cancer (GC) is one of the most common malignancies and the second leading cause of cancer deaths worldwide [1]. It continues to present a major clinical challenge due to delayed diagnosis, limited treatment options, metastasis, and recurrence [2]. Although efforts have been made to improve the survival of GC patients including the use of gastric endoscopy to detect GC earlier, patient compliance to such uncomfortable and invasive procedures is poor. As a result, more than 50% GC patients are already in the progressive stage at first diagnosis, with a 5-year overall survival (OS) rate lower than 30 % [3].

At present, serum carcinoembryonic antigen (CEA) and carbohydrate antigen 72-4 (CA72-4) are the most widely used tumor markers for GC. However, their clinical use is limited by low sensitivity and specificity [4, 5]. Easier, more sensitive, and noninvasive tumor marker assays are urgently needed to improve screening, diagnosis, prognostic evaluation, recurrence monitoring, and follow-up observation of therapeutic efficiency.

Circulating nucleic acids (CNAs), such as circulating cell-free DNA (cf-DNA) and microRNA (miRNA), are extracellular nucleic acids found in serum, plasma, or other body fluids. Many studies [6, 7] have linked CNAs with carcinogenesis and tumor progression. However, the results obtained from different studies are conflicting [8].

LncRNAs are transcripts longer than 200 nucleotides with little or no protein coding function. They are considered a new class of regulatory ncRNAs. Mounting evidence shows that lncRNAs play important roles in a large range of biological processes and diseases, especially in cancer biology [9]. Compared with protein biomarkers expressed in multiple tissue types, lncRNAs often exhibit a tissue-specific expression pattern and can be detected easily in body fluids due to high stability, making them ideal biomarkers [10]. Some lncRNAs, such as HOTAIR [11, 12], H19 [2, 13], GAPLINC [14], ANRIL [15], are reported to be oncogenic molecules in GC. Other lncRNAs act as tumor suppressors, including GAS [16], MEG3 [17, 18], and LEIGC [19]. Yet, whether lncRNAs are expressed in serum and what their potential functions are still elusive.

HULC (highly up-regulated in liver cancer) is overexpressed in liver cancer [20] and can be detected in the plasma of hepatocellular carcinoma (HCC) patients [21]. In the current study, we investigated the serum expression level of HULC in GC patients and explored its clinical value as a serum biomarker for early diagnosis and prognosis prediction of GC.

## RESULTS

### The methodology evaluation of circulating HULC detection in serum samples

**Figure 1:**
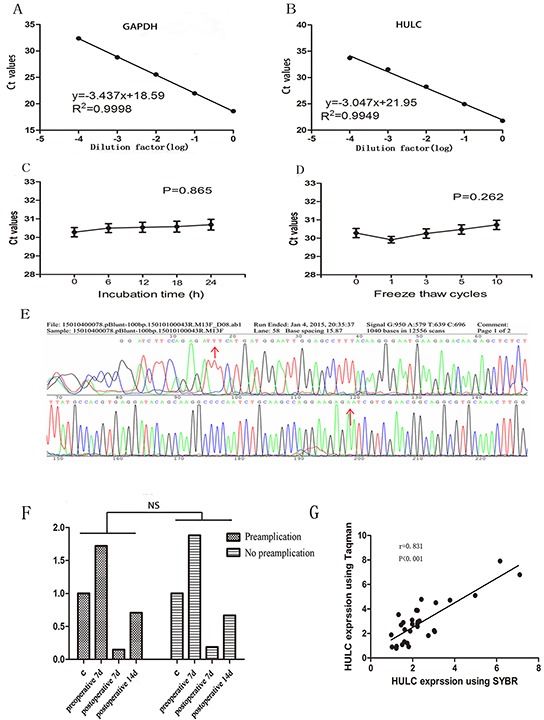
Evaluation of circulating HULC detection methodology **A-B.** Linearity of circulating GAPDH and HULC. Standard curves of serum GAPDH and HULC in a ten-fold serial dilution. **C-D.** Stability of circulating HULC under harsh conditions. (C), prolonged room temperature incubation time or (D), multiple freeze-thaw cycles. Data are presented as raw Ct value (n = 3). **E.** Validation of HULC by Sanger sequencing. **F.** A typical sample with and without pre-amplification showed agreement in tendency. **G.** Spearman's rank correlation scatter plot of HULC levels using SYBR and Taqman probe. Data are presented as relative fold change of lncRNAs. NS means no statistically significant difference.

Due to the relatively non-invasive sampling nature, body fluids are ideal samples for disease diagnosis. To date, no consensus reference has been selected for the normalization of lncRNA levels in body fluids. We selected several possible reference genes beta-actin (ACTB), glyceraldehyde-3-phosphate dehydrogenase (GAPDH), beta-2-microglobulin (β2M), and ribosomal and 18S ribosomal RNA (18SrRNA) for comparison by RT-qPCR in 30 samples (biological replicates). Because glyceraldehyde-3-phosphate dehydrogenase (GAPDH) had good linearity and reproducibility without being affected by age, sex, and pathologic grade (Figure 1A & Supplementary Table S1), it was used as the reference. Then, we evaluate the linearity of HULC using serial ten-fold dilutions of HULC cDNA. The R^2^ of the HULC standard curve was 0.9949, and the regression equation was y = −3.047x+21.95, indicating that qPCR could be used to detect different concentrations of serum HULC (Figure 1B). The intra-assay coefficient of variation (CV) and the inter assay CV of HULC were also satisfactory (Supplementary Table S1). In addition, these sample aliquots were maintained at room temperature (RT) for 0, 6, 12, 18, and 24 hr or were frozen (at −80°C) and thawed (at RT) 0, 1, 3, 5, and 10 times in nuclease-free tubes before processing for RNA isolation. As shown in Figure 1C and 1D, serum HULC levels did not change significantly (P < 0.05), remaining stable under these harsh conditions. Additionally, the PCR products were further detected by sequencing analyses. As expected, their sequences were completely consistent with those from the NCBI database (Figure 1E).

Many laboratories use pre-amplification to improve sensitivity [22]. In the present study, we found that although pre-amplification improved the raw Ct value compared with the conventional fluorescence quantitative PCR assay, there was no significant difference in relative expression (Figure 1F). Other studies used the Taqman technology to improve specificity [23]. In the present study, we used SYBR Green and hydrolysis probes to monitor real-time PCR in 20 randomly chosen samples, and found a significant correlation between our method with conventional RT-qPCR assay and the Taqman method in detecting the relative expression of HULC (r = 0.831; Figure 1G).

### The origin of circulating HULC

**Figure 2:**
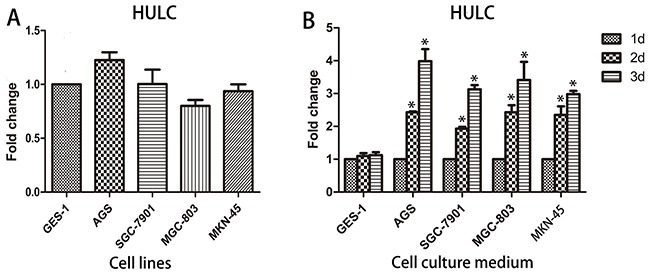
Origin of circulating HULC **A.** The expression of HULC in cell lines showed no statistically significant difference. **B.** HULC was secreted into the cell culture medium in a time-dependent manner. Data are presented as relative fold change of lncRNAs. Single asterisk indicates P < 0.05.

The origin of circulating lncRNA remains a mystery at present. In our study, we found no significant difference in the expression of HULC in four GC cell lines AGS, SGC-7901, MGC-803, and MKN-45 as compared with GES-1 (Figure 2A). Interestingly, we found that the expression of HULC in the culture medium of these GC cell lines increased in a time-dependent manner while no significant change was observed in the cell culture medium of GES-1 (Figure 2B). Based on this phenomenon, we determined that serum lncRNAs may be secreted or leak from tumor cells into the blood circulatory system.

### Increased serum HULC in GC patients

**Figure 3:**
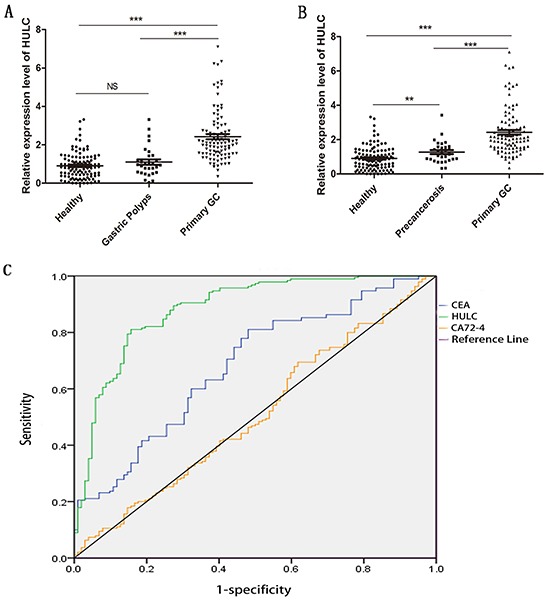
RT-qPCR and ROC curve analysis for predicting HULC as a GC diagnosis biomarker **A.** Scatter plots of serum HULC levels from primary tumor (n = 100), polyps (n = 30), and healthy patients (n = 110). **B.** Scatter plots of serum HULC levels from primary tumor (n = 100), precancerosis (n = 30), and healthy patients (n = 110). Triple asterisks indicate P < 0.001. Double asterisks indicate P < 0.01. NS means no statistically significant difference. **C.** ROC-AUC to compare the diagnostic performance of HULC, CEA, and CA72-4 to discriminate GC from normal controls.

To further validate the interaction between GC and HULC, RT-qPCR analysis was performed to determine the expression level of HULC in 100 primary GC patients, 30 polyp patients, and 110 normal controls. The results showed that serum HULC was highly expressed in GC patients, as compared with the polyps and normal groups (P < 0.001) (Figure 3A). The mean expression level in the GC group was 2.69 times as high as that in normal controls. There was no statistically significant difference between the polyp and normal control groups (P = 0.206). On the basis of these findings, we further explored serum HULC expression levels in various stages of gastric carcinogenesis. Thirty patients with high atypical hyperplasia or intestinal metaplasia as gastric precancerosis were recruited. Compared with precancerosis, serum HULC expression was significantly increased in GC patients (P < 0.001) (Figure 3B). In addition, serum HULC levels differed significantly between precancerosis and normal controls (P < 0.05).

### Diagnostic utility of serum HULC, CEA, and CA72-4 in GC patients

To investigate the characteristics of serum HULC as a potential tumor marker for GC, receiver operating characteristics (ROC) curves were constructed on data from all subjects, including 100 GC patients and 110 healthy controls. Serum HULC effectively differentiated primary GC patients from normal controls with an area under the ROC curves (AUC) of 0.888 (95% confidence interval [CI]: 0.843–0.934; P < 0.001) *vs.* 0.694 (95% CI: 0.621–0.767; P < 0.001) for CEA and 0.514 (95% CI: 0.433–0.595: P = 0.737) for CA72-4 (Figure 3C). At the cutoff value, Youden index was 0.656, the sensitivity was 82%, and the specificity was 83.6%. And combination of HULC and CEA, HULC and CA72-4, or HULC, CEA, and CA72-4 significantly improved the diagnostic sensitivity (Table 2).

**Table 1:** Correlation between HULC expression and clinicopathologic features of GC patients

Characteristic	Total	HULC (n%)		χ2 value	P value
		Low n=52	High n=48		
Gender				0.254	0.615
Male	65	35 (53.8)	30 (46.2)		
Female	35	17 (48.6)	18 (51.4)		
Age (years)				0.098	0.754
≤ 60	38	19 (50.0)	19 (50.0)		
> 60	72	33(53.2)	29(46.8)		
Location				1.767	0.413
Cardia	17	8 (47.1)	9 (52.9)		
Body	9	3 (33.3)	6 (66.7)		
Antrum	74	41 (55.4)	33 (45.6)		
Size (cm)				10.470	0.001**
≥ 5cm	25	6 (24.0)	19 (76.0)		
< 5cm	75	46 (61.3)	29 (38.7)		
Histological differentiation				2.596	0.273
Well	5	4 (80.0)	1 (20.0)		
Moderate	37	21 (56.7)	16 (43.3)		
Poor	58	27 (46.6)	31 (53.4)		
TNM stage				15.204	0.002**
I	23	18 (78.3)	5 (21.7)		
II	21	12 (57.1)	9 (42.9)		
III	45	21 (46.7)	24 (53.3)		
IV	11	1 (9.10)	10 (90.9)		
Lymph node metastasis				5.343	0.021*
Yes	59	25 (59.3)	34 (40.7)		
No	41	27 (38.3)	14 (61.7)		
Distant metastasis				5.663	0.017*
Yes	11	2 (18.2)	9 (81.8)		
No	89	50 (56.2)	39 (43.8)		
*H.pylori* infection				3.983	0.046*
Yes	12	3 (25.0)	9 (75.0)		
No	88	49 (55.7)	39 (44.3)		
CEA				0.002	0.969
Positive	21	11 (52.4)	10 (47.6)		
Negative	79	41 (51.9)	38 (48.1)		
CA72-4				0.003	0.953
Positive	8	4 (50.0)	4 (50.0)		
Negative	92	47 (52.0)	45 (48.0)		

Statistical analyses were carried out using Pearson χ2 test.

* P<0.05 was considered significant

**Table 2:** Use of HULC, CEA and CA72-4 levels to distinguish GC patients from healthy participants

	SEN	SPE	ACCU	PPV	NPV
**CEA**	79.0% (79/100)	53.6% (59/110)	65.7% (138/210)	60.8% (79/130)	73.8% (59/80)
**CA72-4**	66.0% (66/100)	41.8% (46/110)	53.3% (132/210)	50.8% (66/130)	57.5% (46/80)
**HULC**	82.0% (82/100)	83.6% (92/110)	82.9% (174/210)	82% (82/100)	83.6% (92/110)
**HULC+CEA**	94.0% (94/100)	48.2% (53/110)	70.0% (147/210)	62.3% (94/151)	89.8% (53/59)
**HULC+CA72-4**	95.0% (95/100)	37.3% (41/110)	64.8% (136/210)	57.9% (95/164)	89.1% (41/46)
**HULC+CEA+CA72-4**	99.0% (99/100)	46.4% (24/110)	58.6% (123/210)	53.5% (99/185)	96.0% (24/25)

The area under curve (AUC) is shown together with the sensitivity (SEN), specificity (SPE), overall accuracy (ACC), positive predictive value (PPV) and negative predictive value (NPV).

### Correlation between serum HULC expression and clinicopathological parameters of GC

The clinicopathological features of the 100 GC patients are summarized in Table 1. According to the relative expression of HULC in serum, the 100 GC patients were equally classified into two groups: relative high group (n = 48, fold change ≥ 2) and relative low group (n = 52, fold change < 2). High serum HULC expression levels were significantly associated with tumor size (P = 0.001), lymph node metastasis (P = 0.021), distant metastasis (P = 0.017), and tumor-node-metastasis stage (P = 0.002). Noticeably, serum HULC expression was significantly correlated with *H. pylori* infection (P = 0.046). We did not find any correlation between serum HULC expression level and other clinicopathological parameters, such as gender, age, and serum CEA level.

### Dynamic monitoring of serum HULC in GC patients

**Figure 4:**
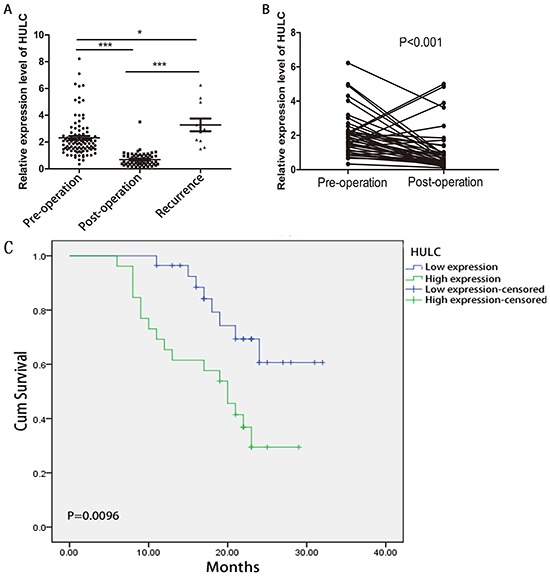
RT-qPCR and Kaplan-Meier analysis for predicting HULC as a GC prognosis biomarker **A.** Scatter plots of serum HULC levels from pre-operative (n = 100), post-operative (n = 62), and recurrent patients (n = 11). **B.** Line chart of serum HULC levels monitored in the 40 surgical GC patients. **C.** Kaplan-Meier survival curve of patients with GC based on HULC expression levels. Patients in the high expression group (n = 27) had significantly poorer prognosis than those in the low expression group (n = 27) (P < 0.05).

Next, we investigated the expression of HULC in 100 primary GC patients, 62 surgical patients, and 11 recurrent patients. The level of serum HULC was significantly lower in the surgical patients than in the primary GC patients and recurrent patients (P < 0.001) (Figure 4A). Pre- and post-operative HULC were also compared in samples from 40 individual patients taken before surgery and at follow-up. As shown in Figure 4B, serum HULC dynamically varied in these patients. Serum HULC decreased over time in most samples except four recurrent patients. The expression of HULC in these four increased sharply one month after surgical treatment and postoperative adjuvant chemotherapy. These data suggest that elevated serum HULC predicts a worse prognosis for GC patients.

### The prognostic value of HULC expression in GC

The prognostic value of HULC expression was investigated using the Kaplan-Meier method. As shown in Figure 4C, there was a significant correlation between HULC expression and OS of GC patients (P < 0.001, log-rank test). The OS rate of GC patients with high HULC expression was significantly lower than that of those with low HULC expression.

## DISCUSSION

In recent years, genome-wide transcriptional studies have indicated that only approximately 2% of the human genome produces biologically meaningful RNA transcripts. A much larger proportion of the genome is transcribed into non-coding RNAs (ncRNAs) [24], 80% of which are mRNA-like lncRNAs transcripts. The deregulated expression of lncRNAs plays a functional role in a variety of physiological processes and disease states [25]. Interestingly, these lncRNAs may be biomarkers for clinical diagnosis and treatment of cancer [26]. Prostate cancer gene 3 (PCA3) is the first prominent example of lncRNAs as a novel biomarker. The noninvasive method to detect PCA3 transcript in urine has outperformed PSA in both sensitivity and specificity to detect prostate malignancy [27, 28]. Arita et al. confirmed the existence of circulating lncRNA H19 in plasma of GC patients [23]. Shao et al. found that AA174084 was aberrantly expressed in gastric juice [29]. All of these findings suggest that circulating lncRNAs have potential value in cancer diagnosis and prognosis prediction.

LncRNA HULC, a liver cancer-associated lncRNA mapped to chromosome 6p24.3 [20], was found to be generally up-regulated in HCC tissues compared with normal liver specimens [20, 21]. Du et al. [30] found that the HULC promoted liver cancer cell proliferation by inhibiting P18. P18, known as eukaryotic translation elongation factor 1 (EEF1E1), can inhibit cell proliferation both *in vivo* and *in vitro*. HULC has obvious induced effects on other digestive system carcinomas, such as GC and pancreatic cancer [31, 32]. Zhao et al. [32] reported that silencing of HULC effectively reversed the epithelial-to-mesenchymal transition (EMT) phenotype, while overexpression of HULC induced patterns of autophagy in GC SGC7901 cells.

Our study is the first to quantify HULC in human serum by using conventional RT-qPCR with good linearity, specificity and reproducibility. We demonstrated that serum HULC expression is significantly higher in primary GC patients than in normal controls and individuals with gastric polyps, while there was no significant difference between the latter two groups. We also found that HULC levels underwent dynamic changes as the disease progressed from a precancerous lesion to cancer. Moreover, serum HULC was significantly decreased in the post-treatment patients to a level that was similar to that in healthy individuals. In addition, the expression of serum HULC quickly rebounded in patients with a collapsed condition even after surgical treatment. Our results show that the level of HULC expression was associated with adverse prognostic factors, and intriguingly we found that the serum HULC level in the *H. pylori* (*HP*)-positive group was higher than that in the *HP*-negative group (P = 0.046). Large-scale epidemiological studies have confirmed that *HP* is a strong risk factor for both GC development and progression. These findings indicate that HULC may be involved in the pathogenesis of GC. Furthermore, ROC to evaluate the diagnostic utility of HULC indicates that serum HULC provides a more powerful differential ability than CEA and CA72-4, suggesting that serum HULC could serve as a promising tumor marker for GC diagnosis. Finally, to investigate the prognostic role of HULC on GC, we performed Kaplan-Meier analysis indicating that HULC could be a marker for outcomes prediction.

In conclusion, our results suggest that increased serum HULC could be an ideal tumor biomarker for GC detection. Simultaneously, serum HULC has potential clinical value for forecasting prognosis and predicting the therapeutic efficacy for GC.

Recent studies have demonstrated that CNAs are detectable in plasma and serum of cancer patients with dysregulation panels [6, 33, 34]. Because blood cells and hemolysis can alter circulating lncRNAs levels [35, 36], we chose to analyze HULC in serum, which is more easily accessible for pre-analytical variables and free of blood cell contamination and the effect of hemolysis.

We observed that serum HULC is surprisingly stable. The underlying mechanism of circulating lncRNAs’ stability in the presence of large numbers of RNases remains unclear. As our study was a preliminary report on the clinical value of circulating HULC in GC, further studies should focus on large-scale sample collection, long-term follow-up, and in-depth functional investigation in order to explore the potential value of HULC in GC.

## MATERIALS AND METHODS

### Study subjects

Serum samples from 173 GC patients, 30 patients with gastric polyps, 30 patients with high atypical hyperplasia or intestinal metaplasia, and 110 age-matched healthy controls (63 males and 47 females; mean age 61 years, range 43-80) were collected. In addition, additional 60 normal samples were recruited to mix together as control in each qPCR experiment. The 173 GC patients consisted of 100 patients with primary GC who had not received any treatment before surgery, 62 surgical patients, and 11 recurrent patients. Forty of the 62 GC patients who received surgery were followed up for one month. All blood samples of the patients with GC, precancerous lesions, and polyps were obtained from the Departments of General Surgery and Gastroenterology at the Affiliated Hospital of Nantong University (Nantong, China) between July 2012 and July 2014. The 170 normal controls were selected from volunteer blood donors of Nantong Blood Center who had no history of autoimmune disease, tissue injury, or trauma at the time of examination. The diagnosis of all 173 GC patients was confirmed by pathology. Histologic grading was performed according to the tumor-node-metastasis (TNM) staging system of the American Joint Committee on Cancer (AJCC) Clinical Practice Guidelines for Oncology (2011).

All samples from 231 males and 161 females with a mean age of 60 years (range 26-85) were anonymous with written informed consent. The study was approved by the Human Research Ethics Committee of the Affiliated Hospital of Nantong University.

### Sample collection

Blood samples were collected in separating gel vacuum collection tubes and centrifuged at 3000×g for 10 min. The upper-layer supernatant was immediately stored in an RNase-free Eppendorf tube at −80°C until use.

### Serum RNA extraction and cDNA synthesis

Serum RNA was extracted from 400 μl serum using Ambion mirVana PARIS kit (Life Technology, USA) according to the manufacturer's protocol. The RNA concentration and purity were measured with a spectrophotometer (NanoPhotometer^TM^, IMPLEN, GER). Complementary DNA was extracted from 10 μg total RNA using the RevertAid First Strand cDNA Synthesis Kit (Thermo Scientific, MA, USA). The mix was incubated at 42°C for 60 min and 70°C for 5 min. The reverse transcription products were stored at −20°C until use for RT-qPCR.

### Quantitative real-time polymerase chain reaction (RT-qPCR)

RT-qPCR was performed on Applied Biosystems 7500 (Applied Biosystems, CA, USA) with 3.0 μl cDNA (with three technical replicates) and 10.0 μl FastStart Universal SYBR Green Master Mix (Roche, GER). The PCR cycling program consisted of incubation for enzyme activation at 95°C for 10 min, followed by denaturation at 95°C for 10 s, annealing at 60°C for 30 s, and then extension at 72°C for 30 s, for a total of 45 cycles. The expression levels of HULC were normalized to the internal control GAPDH to obtain the relative threshold cycle (ΔCT) and mixed normal sera were used as control to calculate relative expression levels (2^−ΔΔCT^). The primers used were as follows: HULC-forward 5′-TCATGATGGAATTGGAGCCTT-3′, HULC-reverse 5′-CTCTTCCTGGCTTGCAGATTG-3′; GAPDH-forward 5′-CCCTTCATTGACCTCAACTA-3′, GAPDH-reverse, 5′-TGGAAGATGGTGATGGGATT -3′.

### Cell culture

Human gastric epithelial cell line (GES-1) and gastric cancer cell lines (AGS, SGC-7901, MGC-803, MKN-45) were purchased from the Chinese Academy of Sciences Cell Bank (Shanghai, China). Cells were cultured in 1640 medium (Gibco BRL, NY, USA) with 10% fetal bovine serum (FBS) and 1% antibiotics (both from Gibco, USA) at 37°C in a humidified incubator under 5% CO_2_ condition.

### Detection of CEA and CA72-4 levels

Serum CEA and CA72-4 were measured with ARCHITECT I2000 SR (Abbott, Chicago, USA). The cutoff value used to make a distinction between positive and negative results was 5 ng/ml for CEA and 10 U/ml for CA72-4.

### Statistical analysis

Statistical analysis was performed using the SPSS software package version 20.0 (SPSS Inc., Chicago, USA). Statistical significance was tested by a Mann-Whitney unpaired test analysis of variance or a Chi-square test as appropriate. P < 0.05 was considered statistically significant. Receiver operating characteristic (ROC) curves and area under the ROC curve (AUC) were used to assess the diagnostic value of using HULC and CEA for GC. The figures were partially drawn by GraphPad Prism 5.0 software (Graphpad Software Inc., CA, USA).

## SUPPLEMENTARY TABLE


