# Supplementary material for: Long non-coding RNA HULC as a novel serum biomarker for diagnosis and prognosis prediction of gastric cancer
Source: Oncotarget. 2016 Jun 16;7(32):51763–72. doi: 10.18632/oncotarget.10107 (PMC5239513; doi:10.18632/oncotarget.10107)
Supplement: Supplementary file 1 [file oncotarget-07-51763-s001.pdf]

Long non-coding RNA HULC as a novel serum biomarker for diagnosis and prognosis prediction of gastric cancer

SUPPLEMENTARY TABLE

Supplementary Table S1: Intra-assay and inter-assay repeatability

	HULC	GAPDH
**Intra-assay**		
**Mean±SE**	32.25±0.2944	30.20±0.0785
**CV(%)**	2.89%	0.82%
**Inter-assay**		
**Mean±SE**	32.88±0.5039	30.76±0.2323
**CV(%)**	4.85%	2.39%
